# Blood lipids influence DNA methylation in circulating cells

**DOI:** 10.1186/s13059-016-1000-6

**Published:** 2016-06-27

**Authors:** Koen F. Dekkers, Maarten van Iterson, Roderick C. Slieker, Matthijs H. Moed, Marc Jan Bonder, Michiel van Galen, Hailiang Mei, Daria V. Zhernakova, Leonard H. van den Berg, Joris Deelen, Jenny van Dongen, Diana van Heemst, Albert Hofman, Jouke J. Hottenga, Carla J. H. van der Kallen, Casper G. Schalkwijk, Coen D. A. Stehouwer, Ettje F. Tigchelaar, André G. Uitterlinden, Gonneke Willemsen, Alexandra Zhernakova, Lude Franke, Peter A. C. ’t Hoen, Rick Jansen, Joyce van Meurs, Dorret I. Boomsma, Cornelia M. van Duijn, Marleen M. J. van Greevenbroek, Jan H. Veldink, Cisca Wijmenga, BIOS Consortium, Erik W. van Zwet, P. Eline Slagboom, J. Wouter Jukema, Bastiaan T. Heijmans

**Affiliations:** Molecular Epidemiology section, Leiden University Medical Center, Einthovenweg 20, Leiden, The Netherlands; Department of Genetics, University of Groningen, University Medical Centre Groningen, Broerstraat 5, Groningen, The Netherlands; Department of Human Genetics, Leiden University Medical Center, Einthovenweg 20, Leiden, The Netherlands; Sequence Analysis Support Core, Leiden University Medical Center, Einthovenweg 20, Leiden, The Netherlands; Department of Neurology, Brain Center Rudolf Magnus, University Medical Center Utrecht, Heidelberglaan 100, Utrecht, The Netherlands; Department of Biological Psychology, VU University Amsterdam, Neuroscience Campus Amsterdam, De Boelelaan 1117, Amsterdam, The Netherlands; Department of Gerontology and Geriatrics, Leiden University Medical Center, Einthovenweg 20, Leiden, The Netherlands; Department of Genetic Epidemiology, ErasmusMC, ’s-Gravendijkwal 230, Rotterdam, The Netherlands; Department of Internal Medicine and School for Cardiovascular Diseases (CARIM), Maastricht University Medical Center, P. Debyelaan 25, Maastricht, The Netherlands; Department of Internal Medicine, ErasmusMC, ’s-Gravendijkwal 230, Rotterdam, The Netherlands; Department of Psychiatry, VU University Medical Center, Neuroscience Campus Amsterdam, De Boelelaan 1117, Amsterdam, The Netherlands; BIOS Consortium, Einthovenweg 20, Leiden, The Netherlands; Department of Medical Statistics and Bioinformatics, Leiden University Medical Center, Einthovenweg 20, Leiden, The Netherlands; Department of Cardiology, Leiden University Medical Center, Einthovenweg 20, Leiden, The Netherlands

**Keywords:** DNA methylation, Lipids, Mendelian randomization, Gene expression

## Abstract

**Background:**

Cells can be primed by external stimuli to obtain a long-term epigenetic memory. We hypothesize that long-term exposure to elevated blood lipids can prime circulating immune cells through changes in DNA methylation, a process that may contribute to the development of atherosclerosis. To interrogate the causal relationship between triglyceride, low-density lipoprotein (LDL) cholesterol, and high-density lipoprotein (HDL) cholesterol levels and genome-wide DNA methylation while excluding confounding and pleiotropy, we perform a stepwise Mendelian randomization analysis in whole blood of 3296 individuals.

**Results:**

This analysis shows that differential methylation is the consequence of inter-individual variation in blood lipid levels and not vice versa. Specifically, we observe an effect of triglycerides on DNA methylation at three CpGs, of LDL cholesterol at one CpG, and of HDL cholesterol at two CpGs using multivariable Mendelian randomization. Using RNA-seq data available for a large subset of individuals (N = 2044), DNA methylation of these six CpGs is associated with the expression of *CPT1A* and *SREBF1* (for triglycerides), *DHCR24* (for LDL cholesterol) and *ABCG1* (for HDL cholesterol), which are all key regulators of lipid metabolism.

**Conclusions:**

Our analysis suggests a role for epigenetic priming in end-product feedback control of lipid metabolism and highlights Mendelian randomization as an effective tool to infer causal relationships in integrative genomics data.

**Electronic supplementary material:**

The online version of this article (doi:10.1186/s13059-016-1000-6) contains supplementary material, which is available to authorized users.

## Background

External stimuli, including tobacco smoke [1], prenatal malnutrition [2], and ultraviolet radiation [3], can induce persistent changes in the epigenome. Circulating immune cells are continuously exposed to a range of stimuli present in plasma. Infectious agents or oxidized low-density lipoprotein (LDL) have been shown to affect the epigenome of monocytes, which, as a result, became protected against secondary infections [4] or acquired a pro-inflammatory phenotype [5], respectively. Here, we investigated whether circulating immune cells are primed by blood lipids *in vivo*, a process potentially relevant for the development of atherosclerosis, which is driven by the interaction between lipids and the immune system [6].

Various population studies have reported on the association between inter-individual variation in blood lipid levels and genome-wide DNA methylation, a key component of the epigenome, in circulating immune cells [7–9]. Although sometimes interpreted as a causal effect of DNA methylation on lipid levels [7], such epigenome-wide association studies (EWASs) cannot distinguish cause and consequence [10]. Large genome-wide association studies (GWASs), however, yielded sets of single nucleotide polymorphisms (SNPs) that are robustly associated with lipid levels [11]. Since genetic variants cannot be the consequence of phenotypic variation, lipid-associated SNPs can serve as causal anchors to test whether elevated lipid levels induce DNA methylation changes in immune cells using Mendelian randomization (MR) [12–14].

To study whether blood lipids can epigenetically prime circulating immune cells, we performed a systematic MR analysis of triglycerides (TGs), LDL cholesterol (LDL-C), and high-density lipoprotein (HDL) cholesterol (HDL-C) levels using whole blood samples of 3296 individuals to interrogate the causal relationship between lipid levels and DNA methylation, while excluding confounding and pleiotropy. Using this approach to analyze both DNA methylation and transcriptome data from 2044 individuals, we identified specific differences in DNA methylation that are induced by blood lipids and could be linked to expression of genes with a well-established role in lipid metabolism.

## Results

The association between blood lipids and genome-wide DNA methylation in whole blood was investigated in 3296 individuals from six cohorts (Table 1; the six cohorts are in the Biobank-based Integrative Omics Study (BIOS) Consortium, a full list of the authors of which is available in Additional file 1). All cohorts were comprised of men and women (32–60 % men) and the age of the individuals ranged from 18 to 81 years.

**Table 1:** Characteristics of the six cohorts in the BIOS Consortium

	CODAM	LL	LLS	NTR	PAN	RS
Number of individuals	164	748	785	692	184	723
RNA-Seq^a^	159	616	650			619
Gender (% male)	50	42	48	32	60	42
Age (years) [SD]	65.6 [6.8]	45.6 [13.3]	58.4 [7.5]	34 [12.1]	62.4 [9.4]	67.6 [6.0]
TG (mmol/L) [SD]	1.6 [0.8]	1.2 [0.9]	1.9 [1.2]	1.3 [0.7]	1.9 [1.1]	1.5 [0.9]
LDL-C (mmol/L) [SD]	3.6 [1.0]	3.0 [0.9]	3.4 [1.0]	2.8 [0.8]	3.4 [0.9]	3.3 [0.9]
HDL-C (mmol/L) [SD]	1.3 [0.3]	1.5 [0.4]	1.4 [0.4]	1.4 [0.9]	1.4 [0.4]	1.5 [0.4]
Monocytes (%) [SD]	7.9 [1.6]	8.5 [2.2]	5.5 [1.5]	7.7 [2.0]	6.8 [1.1]	7.1 [2.1]
Lymphocytes (%) [SD]	51.2 [14.0]	34.5 [7.4]	29.2 [6.9]	35.1 [7.8]	31.8 [7.1]	36.3 [7.9]
Neutrophils (%) [SD]	40.7 [6.9]	53.3 [8.0]	59.8 [7.7]	52.6 [7.7]	55.3 [7.0]	48.3 [7.1]

^a^The number of individuals for which RNA-Seq data were available

*CODAM* Cohort on Diabetes and Atherosclerosis Maastricht, *LL* LifeLines, *LLS* Leiden Longevity Study, *NTR* Netherlands Twin Register, *PAN* Prospective ALS Study Netherlands, *RS* Rotterdam Study, *SD* standard deviation

**Fig. 1:**
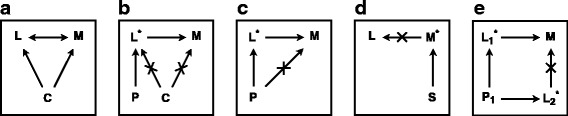
Illustration of the stepwise Mendelian randomization approach. **a** In a conventional EWAS, associations observed are potentially confounded (*C*) and the direction of the association between lipids (*L*) and DNA methylation (*M*) cannot be inferred. **b** Using Mendelian randomization, polygenic scores (*P*) are used to obtain an unconfounded proxy for lipid levels and, since M cannot influence P, an effect of L on M can be inferred. **c** An additional analysis is required to exclude a direct effect of P on M (i.e., *cis*-methylation quantitative trait loci (QTL) effect of polygenic score SNPs) not mediated through L. **d** Reverse causation, i.e., an effect of M on L, is excluded by evaluating the association of local genetic variation (*S*) affecting M (*cis*-methylation QTL) on lipid levels. **e** Pleiotropic effects are excluded using a multivariate model that incorporates all lipids and their polygenic scores

First, we performed EWASs of TG, LDL-C, and HDL-C levels for 453,109 CpGs (Fig. 1a and Additional file 2). For TG, we observed 21 differentially methylated CpGs (false discovery rate (FDR)-adjusted *P* value <0.05; Table 2) with effect sizes ranging from −0.9 to +2.4 % change in DNA methylation per standard deviation (SD) difference in TG levels. For LDL and HDL levels, we found three and four differentially methylated CpGs with effect sizes ranging from 0.4–1.0 % per SD and 0.2–0.7 % per SD, respectively. The direction of the observed associations were consistent across the six cohorts (Additional file 3). The EWAS effect size estimates were not sensitive to the exclusion of non-fasted samples (24 % of the individuals), the exclusion of samples with imputed cell counts (15 % of the individuals), or the addition of current smoking behavior, lipid-lowering medication, or body mass index (BMI) as covariates (Additional file 4).

**Table 2:** Associations between log TG, LDL-C, and HDL-C levels and DNA methylation

		Chromosome	Position	Mean DNAm (%)	Estimate (%/SD)^a^	*P*_*FDR*_
TG	cg14476101	1	120,255,993	63	−0.8 (−1.0, −0.6)	2.6 × 10^−8^
	cg19693031	1	145,441,553	73	−0.6 (−0.8, −0.5)	5.2 × 10^−9^
	cg06690548	4	139,162,809	85	−0.6 (−0.7, −0.5)	5.7 × 10^−12^
	cg05575921	5	373,379	79	−0.9 (−1.3, −0.6)	4.3 × 10^−3^
	cg14817490	5	392,921	32	−0.5 (−0.6, −0.3)	3.3 × 10^−2^
	cg06560379	6	44,231,306	12	−0.2 (−0.2, −0.1)	4.0 × 10^−2^
	cg19589396	8	103,937,375	69	−0.4 (−0.5, −0.2)	7.6 × 10^−3^
	cg07504977	10	102,131,013	41	0.5 (0.3, 0.6)	2.4 × 10^−4^
	cg11376147	11	57,261,199	21	−0.2 (−0.3, −0.1)	7.3 × 10^−3^
	cg00574958	11	68,607,623	12	−0.4 (−0.5, −0.3)	2.4 × 10^−23^
	cg17058475	11	68,607,738	14	−0.4 (−0.5, −0.3)	7.9 × 10^−7^
	cg12556569	11	116,664,040	26	2.4 (1.5, 3.4)	1.4 × 10^−2^
	cg15863539	17	17,716,951	79	0.2 (0.1, 0.3)	1.3 × 10^−3^
	cg20544516	17	17,717,184	73	0.3 (0.2, 0.4)	1.1 × 10^−3^
	cg11024682	17	17,730,095	46	0.5 (0.3, 0.6)	3.8 × 10^−11^
	cg08857797	17	40,927,700	57	0.4 (0.3, 0.6)	7.3 × 10^−3^
	cg03636183	19	17,000,586	68	−0.6 (−0.8, −0.3)	1.3 × 10^−2^
	cg02711608	19	47,287,965	24	−0.3 (−0.4, −0.2)	3.9 × 10^−2^
	cg27243685	21	43,642,367	83	0.4 (0.4, 0.5)	8.4 × 10^−20^
	cg01881899	21	43,652,705	13	0.3 (0.2, 0.3)	1.7 × 10^−4^
	cg06500161	21	43,656,588	60	0.7 (0.6, 0.8)	1.5 × 10^−29^
LDL-C	cg27168858	1	55,351,660	78	0.4 (0.3, 0.6)	5.3 × 10^−3^
	cg00908766	1	109,817,497	59	1.0 (0.6, 1.3)	2.3 × 10^−2^
	cg05119988	4	166,251,190	59	0.5 (0.3, 0.6)	1.4 × 10^−4^
HDL-C	cg17901584	1	55,353,707	53	0.7 (0.5, 0.9)	1.2 × 10^−4^
	cg26313301	19	11,219,616	87	−0.2 (−0.3, −0.1)	3.6 × 10^−2^
	cg27243685	21	43,642,367	83	−0.4 (−0.5, −0.3)	1.2 × 10^−9^
	cg06500161	21	43,656,588	60	−0.7 (−0.8, −0.5)	1.3 × 10^−13^

^a^Estimate is the percentage change in DNA methylation per standard deviation change in lipid levels

*DNAm* DNA methylation

EWASs are prone to bias due to unmeasured confounding and, crucially, cannot be used to infer whether the associations arise from an influence of lipids on DNA methylation or DNA methylation on lipids. To address these limitations, we performed a MR analysis to determine causality while excluding confounding (Fig. 1b). We constructed weighted polygenic scores (PSs) for TG, LDL-C, and HDL-C levels (Additional file 5) using genetic variants identified in a large-scale meta-analysis of GWASs of lipid levels [11] and evaluated their validity in our own data (i.e., an F-statistic >10 and no association with known confounders). The PSs for TG (30 SNPs), LDL-C (28 SNPs), and HDL-C (60 SNPs) explained a relatively small, but highly significant, proportion of the variance (5.6 % [F = 157, *P* = 1.7 × 10^−40^], 3.1 % [F = 99, *P* = 6.2 × 10^−27^] and 5.1 % [F = 164, *P* = 1.1 × 10^−36^]; Additional file 6) and were not associated with confounders (Additional file 7) with the exception of the HDL-C PS, which was associated with confounder neutrophil counts (*P* value = 1.8 × 10^−2^). However, the level of statistical significance was very weak when compared with the association between HDL-C PS and HDL-C levels. Using the PSs as unbiased predictors of lipids levels, we confirmed an effect of TG on 9 out of 21 EWAS-identified CpGs (*P* value <0.05; Additional file 8). For LDL-C and HDL-C, an effect was found for two of the three and two of the four CpGs, respectively. Since the CpGs with higher *P* values (i.e., lowest level of statistical evidence) in the EWAS analysis were the ones that did not survive the MR approach, the multiple testing correction applied in the EWAS step (*P*_*FDR*_ <0.05) for the association with lipid levels remains applicable to the MR-identified CpG set. Interestingly, MR and EWAS estimates for the effect sizes were highly concordant for the EWAS-identified CpGs, also for CpGs not statistically significant in the MR step. The confidence intervals of the MR estimates were wider, however, in line with the moderate proportion of variance explained by the PSs (Additional file 9). Post hoc power calculations [15] were performed for three scenarios where the EWAS effect size estimate is (1) the true causal effect size, (2) half the true causal effect size, and (3) double the true causal effect size (Additional file 10). The power estimates indicated that the causal effects we identified in our MR analysis were the ones for which we had the highest statistical power.

SNPs in the PS may directly influence DNA methylation (i.e., a methylation QTL effect) [16], violating the assumption of MR that the effect is mediated through lipid levels (Fig. 1c). Of the MR-identified CpGs, three mapped within 1 Mbp of a PS SNP, for which analyses were re-run with the SNP added as covariate to the model (Additional file 11). The association of CpGs cg12556569 with TG and cg00908766 with LDL-C was mediated by a direct effect of a PS SNP *in cis* (rs964184 [mapping 15,121 bp from the CpG] and rs629301 [808 bp from the CpG]). The association of CpG cg27168858 with LDL-C, however, was not explained by a nearby PS SNP (rs2479409 [152,989 bp from the CpG]). Thus, for TG, LDL-C, and HDL-C, eight, one and two CpGs remained, respectively. Of interest, CpGs cg06500161 and cg27243685 were associated with both TG and HDL-C.

Next, we evaluated the possibility that DNA methylation affected lipid levels, i.e., the reverse of the association implied by the MR analysis. To this end, for every MR-identified CpG we established the SNP *in cis* (<100 kb) with the strongest association with methylation level defined as the lowest *P* value (Additional file 12) and used this SNP as a proxy for DNA methylation (Fig. 1d; Additional file 13). We found no evidence of an effect of DNA methylation on lipid levels at these CpGs. In addition to the MR-identified CpGs, we performed the same analysis for all EWAS-identified CpGs (Additional files 12 and 13). Two SNPs could not be evaluated because the SNP was either located in the CpG site (cg12556569) or was in linkage disequilibrium (R^2^ = 0.52) with a PS SNP (cg00908766), violating MR assumptions. For all the other six CpGs, no effect of DNA methylation on lipid levels was found. Thus, no evidence for an effect on lipid levels was observed for any of the CpGs (either surviving the previous MR steps or identified in the initial EWAS analysis) and reverse causation is, therefore, unlikely.

A final limitation precluding an unequivocal interpretation of the analysis so far is the possibility of pleiotropy. The SNPs used to construct PSs are primarily, but not solely, associated with their respective lipid level [11]. Using a multivariable MR analysis with all three lipids and their PSs (Fig. 1e), we found that the two CpGs overlapping between TG and HDL-C were driven by HDL-C while their association with TG could be attributed to pleiotropy (Table 3). For an additional three CpGs associated with TG, pleiotropic effects could not be formally excluded (*P* value >0.05). The effect of TG, LDL-C, and HDL-C on the remaining three, one, and two CpGs, respectively, was not driven by pleiotropic effects. To exclude the occurrence of unmeasured and unknown pleiotropic effects, we applied Egger regression [17]. This analysis showed that there was no net pleiotropic effect (Table 4). However, while multivariable MR indicated that the association for the two CpGs overlapping between TG and HDL-C could be attributed to an effect of HDL-C, Egger regression indicated that it was due to TG. Table 5 summarizes the outcome from the sequential steps of our stepwise MR approach on EWAS-identified CpGs.

**Table 3:** Estimated effect of TG, LDL-C, and HDL-C levels on DNA methylation using a multivariable MR analysis to exclude pleiotropic effects

		Chromosome	Position	Mean DNAm (%)	Estimate (%/SD)^a^	*P* value
TG	cg00574958	11	68,607,623	12	−0.7 (−1.2, −0.3)	1.5 × 10^−3^
	cg17058475	11	68,607,738	14	−1.0 (−1.6, −0.3)	2.5 × 10^−3^
	cg11024682	17	17,730,095	26	1.0 (0.3, 1.7)	5.4 × 10^−3^
LDL-C	cg27168858	1	55,351,660	78	1.4 (0.5, 2.3)	3.1 × 10^−3^
HDL-C	cg27243685	21	43,642,367	83	−0.7 (−1.1, −0.3)	2.2 × 10^−3^
	cg06500161	21	43,656,588	60	−1.1 (−1.8, −0.5)	3.5 × 10^−4^

^a^ *Estimate* is the percentage change in DNA methylation per standard deviation change in lipid levels

*DNAm* DNA methylation

**Table 4:** Estimated effect of TG, LDL-C, and HDL-C levels on DNA methylation using an approach based on Egger regression to exclude pleiotropic effects, including *P* values for an estimate of net pleiotropic effects of the PS SNPs

		Chr	Position	Mean DNAm (%)	Estimate (%/SD)^a^	*P* value	Pleiotropy *P* value
TG	cg00574958	11	68,607,623	12	−1.1 (-1.4, -0.7)	5.7 × 10^−9^	0.37
	cg17058475	11	68,607,738	14	−1.3 (-1.8, -0.8)	2.3 × 10^−7^	0.82
	cg11024682	17	17,730,095	26	0.9 (0.3, 1.5)	1.9 × 10^−3^	0.50
	cg27243685	21	43,642,367	83	0.7 (0.3, 1.1)	6.4 × 10^−4^	0.18
	cg06500161	21	43,656,588	60	1.4 (0.8, 2.0)	2.9 × 10^−6^	0.75
LDL-C	cg27168858	1	55,351,660	78	0.7 (0.1, 1.3)	3.1 × 10^−2^	0.36
HDL-C	cg27243685	21	43,642,367	83	0.3 (-0.9, 1.5)	0.62	0.79
	cg06500161	21	43,656,588	60	−0.1 (-1.8, 1.7)	0.93	0.15

^a^ *Estimate* is the percentage change in DNA methylation per standard deviation change in lipid levels

*DNAm* DNA methylation

**Table 5:** Overview of the stepwise Mendelian randomization approach

		b	c	d	e
TG	cg14476101	✗			
	cg19693031	✓	✓	✓	✗
	cg07504977	✓	✓	✓	✗
	cg11376147	✗			
	cg00574958	✓	✓	✓	✓
	cg17058475	✓	✓	✓	✓
	cg12556569	✓	✗		
	cg15863539	✗			
	cg20544516	✗			
	cg11024682	✓	✓	✓	✓
	cg08857797	✗			
	cg03636183	✗			
	cg02711608	✗			
	cg27243685	✓	✓	✓	✓^1^
	cg01881899	✓	✓	✓	✗
	cg06500161	✓	✓	✓	✓^1^
	cg06690548	✗			
	cg05575921	✗			
	cg14817490	✗			
	cg06560379	✗			
	cg19589396	✗			
LDL-C	cg27168858	✓	✓	✓	✓
	cg00908766	✓	✗		
	cg05119988	✗			
HDL-C	cg17901584	✗			
	cg26313301	✗			
	cg27243685	✓	✓	✓	✓^2^
	cg06500161	✓	✓	✓	✓^2^

Starting from associations between lipid levels and DNA methylation, a causal and unconfounded effect of lipid levels on DNA methylation was estimated (b), a direct effect of genetic variants on DNA methylation was excluded (c), reverse causation was excluded (d) and pleiotropy was excluded (e)

A *tick* indicates a CpG passed step; a *cross* indicates a CpG failed step

^1^Egger regression only

^2^Multivariable MR only

The CpGs resulting from the stepwise MR analysis were annotated to blood cell chromatin states [18], which inform on the biological role of the genomic region harboring the CpG. This analysis showed that all CpGs were located in regulatory regions active in blood cells (Table 6). To link the differentially methylated CpGs to a specific gene, we tested for an association between the methylation level of the six CpGs resulting from the MR analysis and gene expression *in cis* (<100 kb from transcription start site (TSS)). Remarkably, all CpGs were associated with gene expression (*P* value <0.05; Table 6; Additional file 14) and, in all cases, it was the gene in which the CpG was located. The TG-influenced CpGs cg00574958 and cg17058475 (located in a region flanking an active TSS and in an enhancer, respectively) were both associated with expression of *CPT1A* and CpG cg11024682 (located in an enhancer region) was linked to *SREBF1* expression. The LDL-C-influenced CpG cg27168858 (located in an active TSS flanking region) mapped to *DHCR42*. Finally, the TG- (Egger regression) or HDL-C- (multivariable MR) influenced CpGs cg06500161 and cg27243685 (located in a region of weak transcription and flanking an active TSS, respectively) were associated with *ABCG1* expression. All these genes have a central role in lipid metabolism.

**Table 6:** DNA methylation of MR-identified CpGs was associated with gene expression of genes involved in lipid metabolism

		Chr	Position	Mean DNAm (%)	Gene	Estimate (logCPM/β)^a^	*P* value	Chromatin state
TG	cg00574958	11	68,607,623	12	*CPT1A*	−3.3 (−4.1,−2.5)	1.5 × 10^−14^	Flanking active TSS
	cg17058475	11	68,607,738	14	*CPT1A*	−1.7 (−2.3,−1.1)	1.8 × 10^−8^	Enhancer
	cg11024682	17	17,730,095	26	*SREBF1*	−2.4 (−2.8,−2.0)	5.2 × 10^−33^	Enhancer
LDL-C	cg27168858	1	55,351,660	78	*DHCR24*	−4.1 (−4.9,−3.3)	2.4 × 10^−25^	Flanking active TSS
HDL-C	cg27243685	21	43,642,367	83	*ABCG1*	−4.5 (−5.4,−3.6)	4.0 × 10^−24^	Flanking active TSS
	cg06500161	21	43,656,588	60	*ABCG1*	−5.1 (−5.7,−4.5)	1.2 × 10^−56^	Weak transcription

CpGs are located in regions of active gene regulation

^a^ *Estimate* is the log counts per million change in gene expression per β (0–1) change in DNA methylation

*DNAm* DNA methylation

## Discussion

We performed EWASs of blood lipids in a meta-analysis comprising 3296 individuals followed by a stepwise MR approach to evaluate the association of blood lipids with DNA methylation in circulating immune cells and infer the direction of causality. Our analysis showed that differential methylation was induced by TG at three CpGs, by LDL-C at one CpG, and by either TG or HDL-C at two CpGs. We did not observe evidence for the reverse relationship, that is, an effect of DNA methylation on lipid levels. These data indicate that blood lipids can epigenetically prime immune cells, which may prove relevant for disease phenotypes with an inflammatory component [4, 5].

All the CpGs we identified mapped to regions with active regulatory roles in blood cells and were associated with the expression of genes with a central role in lipid metabolism. Higher TG levels induced lower methylation of CpGs cg00574958 and cg17058475, which was associated with higher expression of *CPT1A.* Higher TG levels also induced higher methylation of CpG cg11024682, which was associated with lower expression of *SREBF1. CPT1A* attaches carnitine to long-chain fatty acids, which is required for entry into the mitochondria and subsequent catabolism [19]. *SREBF1* regulates energy homeostasis, including genes involved in the synthesis, import, and efflux of lipids. Sterols, such as cholesterol, inhibit the effect of *SREBF1* [20]. Higher LDL-C levels induced higher methylation of a CpG in *DHCR24*, which was linked to lower *DHCR24* expression. *DHCR24* catalyzes the reduction of desmosterol to cholesterol, the last step in the Bloch cholesterol biosynthesis pathway [21]. Either lower TG or higher HDL-C levels induced lower methylation of CpGs cg06500161 and cg27243685, which in turn were associated with higher expression of *ABCG1. ABCG1* mediates cellular cholesterol efflux to HDL in the reverse cholesterol transport pathway. The reverse cholesterol transport pathway transfers cholesterol from peripheral tissues via the blood to the liver [22]. Taken together, higher TG and LDL-C and lower HDL-C levels lead to DNA methylation changes that are associated with, on the one hand, increased catabolism and cellular export and, on the other hand, decreased cellular import of lipids. These findings suggest a role for epigenetic priming in end-product feedback control, a process where the end-product inhibits its own synthesis and that has been observed for cholesterol [23].

Our analysis replicated previous EWASs reporting the association between TG and two CpGs in *CPT1A* in CD4+ T cells [7] and whole blood [8]. In addition, eight out of ten differentially methylated CpGs found in the most recent EWAS of various blood lipids were replicated [9] (7/8 CpGs replicated for TG, 0/1 for LDL-C, 1/1 for HDL-C). The CpGs that we did not replicate were cg20544516 associated with TG (rank 111 in our analysis, *P* = 1 × 10^−6^) and cg22178392 associated with LDL-C (rank 308,850 in our analysis, *P* = 0.68). In contrast to earlier interpretations of these findings [9], our MR analysis indicates that differential methylation is the consequence of inter-individual variation in blood lipid levels instead of its cause. Interestingly, EWASs of lipid levels also overlapped with those of other outcomes. An EWAS of BMI reported differential methylation at *CPT1A* and *ABCG1* [24], and an EWAS of type 2 diabetes at *SREBF1* and *ABCG1* [25]. Our MR analysis using polygenic scores of lipid levels predicts that the altered lipid profile in blood that is associated with BMI and type 2 diabetes was driving these outcomes.

The stepwise MR approach we implemented circumvents various issues commonly encountered in MR. We used a weighted polygenic score (PS) for the MR analysis with weights estimated in a large lipid GWAS [11] to test the hypothesis that lipid levels affected DNA methylation. The PS optimally captured the inter-individual variance in lipid levels and is less prone to violations of MR assumptions compared with single genetic variants [26]. Furthermore, we could exclude the violation of the assumption in MR that genetic variants in the PS have a direct effect on DNA methylation by testing the association of lipid-associated differential methylation with PS SNPs *in cis*. This contrasts with most MR studies where a direct effect of genetic variants on the outcome is unknown [12]. Remaining key issues in MR, namely reverse causation and pleiotropic effects, were addressed using a bidirectional multivariable model [27] and an approach based on Egger regression, which accounts for unmeasured and unknown pleiotropic effects [17]. Together these analyses excluded the occurrence of reverse causation (here an effect of DNA methylation on lipid levels) and an involvement of pleiotropy, supporting a causal role of lipid levels in its association with DNA methylation in circulating immune cells. Surprisingly, multivariable MR and Egger regression yielded opposite results for the two *ABCG1* CpGs that were associated with both TG and HDL-C. While multivariable MR indicated that the association could be attributed to an effect of HDL-C, Egger regression indicated that it was due to TG. These discrepant outcomes may be indicative of the limits of MR analysis in the presence of a substantial correlation between evaluated phenotypes, as is the case for lipid levels. We presume that the result of the multivariable MR analysis is closer to the biological mechanism in this case because the analysis uses the actual measured lipid levels. Moreover, this interpretation is consistent with the biological role of *ABCG1*, which is a key mediator of cellular cholesterol efflux to HDL particles.

We performed our analysis in a large meta-analysis of cohorts with matched genotype and genome-wide DNA methylation data. However, MR analysis has a relatively low power; the lipid PSs capture only about 5 % of the total variance in lipid levels. Therefore, we adopted an approach in which we performed a discovery phase using a regular EWAS approach followed by validation and causal inference using a stepwise MR approach. Only for a subset of EWAS findings were we able to infer the direction of causality in the MR approach. These were the ones for which our study had a relatively high statistical power. However, it was striking that the effect size estimates from the EWASs were generally highly consistent with those from the MR analysis, which is compatible with an effect of lipid levels on DNA methylation for more CpGs. One may argue that individual steps in our MR approach require additional multiple testing. This would have further limited our findings in the multivariable MR approach to those for LDL-C and HDL-C, for which fewer associated CpGs were observed and which are thus less affected by additional multiple testing adjustment. We believe that this would be too conservative, since the association of the lipid levels with DNA methylation of specific CpGs was already corrected for multiple testing in the EWAS. These considerations have two implications. First, larger studies of blood lipid are expected to detect additional CpGs influenced by lipid levels. Second, sample sizes should ideally be increased to achieve sufficient statistical power to directly evaluate the association of PSs with DNA methylation on a genome-wide scale. This would remove the necessity of a discovery phase using EWAS to preserve statistical power and would resolve the issue pertaining to the question of additional adjustment for multiple testing in a stepwise approach. Second, most of the resulting associations will be explained by an effect of blood lipids on DNA methylation. The power to test reverse causal relationships (that is of DNA methylation on blood lipids) can be improved by the availability of comprehensive catalogues of methylation QTL enabling testing both *in cis* and *in trans.*

Cell counts are known confounders in EWASs measuring whole blood [10, 28] and were included as a covariate in the analyses. However, this also impedes the discovery of cell type-specific processes. Hence, it remains to be determined if specific immune cells are particularly prone to epigenetic priming by lipid levels. However, previous EWASs of lipid levels in adipose tissue (cg27243685 and cg11024682) and in skin biopsies (cg11024682) reported differential methylation at CpG sites also identified in whole blood [9]. This suggests that the lipid priming does not occur exclusively in circulating immune cells. A recent study of the TG-lowering drug fenofibrate suggested that a 3-week daily treatment was not sufficient to reverse lipid-associated DNA methylation changes [29] despite the short half-life of various blood cell types. Together, the presence of lipid-induced changes across tissues and the inability to reverse these changes in circulating cells in the short-term open the possibility that these changes did not arise in the circulation but occurred already in hematopoietic stem cells, where lipid priming has previously been observed [30].

## Conclusions

We implemented a systematic MR approach to uncover the occurrence of epigenetic priming on immune cells and show that it can be used effectively when genetic variants for the stimulus of interest are available. Our findings imply that differential methylation observed in EWASs may frequently be a consequence rather than a cause of an outcome of interest. Future studies should establish whether epigenetic priming plays a role in the etiological path from risk factor to disease outcome. We anticipate that with the rapid increase in the availability of genomics, epigenomics, transcriptomics and metabolomics data, approaches like ours will be increasingly used to unravel causal relationships in integrative genomics approaches [14].

## Methods

### Cohorts

The Biobank-based Integrative Omics Study (BIOS) Consortium [31, 32] consists of the six Dutch cohorts Cohort on Diabetes and Atherosclerosis Maastricht (CODAM) [33], LifeLines (LL) [34], Leiden Longevity Study (LLS) [35], the Netherlands Twin Register (NTR) [36], Rotterdam Study (RS) [37], and Prospective ALS Study Netherlands (PAN) [38]. For all 3296 unrelated individuals genotypes, DNA methylation and blood profiles (including lipid levels and cell counts) were measured in whole blood, which was collected simultaneously for all measurements. RNA-Seq data were also available for 2044 individuals. DNA methylation and RNA-Seq data were generated by the Human Genotyping facility (HugeF) of ErasmusMC, the Netherlands (http://www.glimdna.org/). Characteristics of the cohorts can be found in Table 1.

### Lipid measurements and cell counts

Triglyceride (TG), HDL cholesterol (HDL-C), and total cholesterol levels (TC) were measured after a fasting period of 12 h for CODAM, LL, NTR (for 678/692 individuals), RS, and PAN; for LLS non-fasted lipids were measured for 757 of the 785 individuals. LDL cholesterol (LDL-C) was calculated using Friedewald’s method [39]. For all analyses, TG levels were log-transformed and all lipid levels were scaled to have mean 0 and standard deviation 1.

White blood cell counts (WBC), i.e. neutrophils, lymphocytes, monocytes, eosinophils, and basophils, were measured by the standard WBC differential as part of the complete blood count (CBC). However, a minority of samples were lacking CBC measurements (15 %) or did not differentiate between granulocyte subtypes (neutrophils, eosinophils, and basophils; 37 %). Since DNA methylation levels are informative for the white blood cell composition [40], a predictor was built to infer the white blood cell composition of those samples using partial least squares, which can handle both multivariate responses and high-dimensional covariates. The R package *pls* [41] was used to fit the model and to optimize the number of pls components using fivefold cross-validation, including age and gender as covariates. The predictor was trained on two-thirds of the samples with complete WBC data (N = 1364) and tested on one-third (N = 682) of samples across cohorts to obtain a validated predictor. The correlations between measured and predicted leukocyte subtype percentages in the test set were 0.84, 0.86, and 0.68 for neutrophils, lymphocytes, and monocytes, respectively. The R code and detailed documentation for the function *wbccPredictor* are available from https://github.com/mvaniterson/wbccPredictor.

### Genotypes

SNPs were measured per cohort (for data generation details see Tigchelaar et al. [34] for LL, Deelen et al. [42] for LLS, Willemsen et al. [36] for NTR, and Hofman et al. [37] for RS), harmonized (*Genotype Harmonizer* [43]), and imputed (*Impute2* [44]) using GoNL5 [45] as reference. SNPs with an imputation info-score <0.5, Hardy–Weinberg equilibrium *P* value <10^−4^, call rate <95 % or minor allele frequency <0.05 were removed.

### DNA methylation

Using the Zymo EZ DNA methylation kit (Zymo Research, Irvine, CA, USA), 500 ng of genomic DNA was bisulfite-converted and 4 μl of bisulfite-converted DNA was measured on the Illumina 450 k array using the manufacturer’s protocol (Illumina, San Diego, CA, USA). Quality control and normalization of the data were done according to Tobi et al. [46]. In brief, sample outliers were detected and removed using *MethylAid* [47] (95 samples removed), probes with a detection *P* value >0.01, bead number <3, or zero intensity were removed, as well as the ambiguously mapped probes (34,064 probes removed) [48]. This yielded a set of 453,109 CpGs measured in 3296 samples. Next, the DNA methylation data were normalized using the functional normalization [49] approach as implemented in *minfi* [50] (with five principal components of the control probes). Samples where 5 % of the probes failed or probes where 5 % of the samples failed were excluded (0 samples and 0 probes removed). The final dataset consisted of 453,109 CpGs from 3296 individuals. The R code for the quality control and normalization pipeline is available at https://git.lumc.nl/molepi/Leiden450K. 

### Gene expression

Total RNA libraries were generated using the TruSeq v2 library protocol and 2 × 50-bp paired-end sequencing was performed on the Illumina Hiseq2000. Reads passing Illumina’s Chastity filter were produced using *CASAVA* and quality control was done with *FastQC* [51], *cutadapt* [52] (adapter trimming), and *Sickle* [53] (removal of low-quality read ends). Reads were aligned to the human genome (build NCBI37) using *STAR* [54]. Gene quantifications were obtained as the total number of reads that aligned to the exons of a gene as annotated by Ensembl v.71. Subsequently, gene counts were normalized using *cqn* [55] to correct for gene- and sample-specific GC biases. Linear model fitting and inference were performed using *voom* [56] and *limma* [57]. This resulted in gene expression data for 2044 of the 3296 individuals (Table 1).

### Statistical analysis

All analyses were performed using R [58]. For the EWAS of lipid levels, the association of DNA methylation with TG, HDL-C, and LDL-C was evaluated per cohort using the linear model defined by Eq. 1:

1 $$ DNA{m}_i={\beta}_0+{\beta}_1\cdot lipi{d}_i+{\beta}_2\cdot gende{r}_i+{\beta}_3\cdot ag{e}_i+{\beta}_4\cdot \% mon{o}_i+{\beta}_5\cdot \% lymp{h}_i+{\beta}_6\cdot \%neu{t}_i+{\beta}_7\cdot bisplat{e}_i+{\beta}_8\cdot positio{n}_i+{\varepsilon}_i\ \left(i=1,\dots, 3296\right) $$

where %mono, %lymph, and %neut are the percentages of monocytes, lymphocytes, and neutrophils, bisplate is the bisulfite plate number, and position is the position on the 450 k array.

To minimize possible inflation in the test statistic, the genomic control procedure [59] was applied to achieve an inflation factor λ of 1.0. Nominal *P* values and standard errors were calculated from the resulting corrected t-statistics. The outcomes of the six cohorts were combined in a fixed-effect meta-analysis using *metaphor* [60], followed by genomic control on the resulting z-statistics (λ = 1.0). *P* values were calculated from corrected z-values and adjusted for multiple testing using the Benjamini–Hochberg (FDR) procedure.

Polygenic scores (PSs) were constructed from GWAS [11] SNPs to serve as an instrument in MR analysis. For each SNP, dosages were calculated using Eq. 2:

2 $$ Dosage=0\cdot AA+1\cdot AB+2\cdot BB $$

where AA, AB, and BB are the measured allele frequencies.

SNPs primarily associated with their respective lipid level (Additional file 5) were used to calculate the PSs as defined by Eq. 3:

3 $$ PS=\frac{dosag{e}_1\cdot E{S}_1+ dosag{e}_2\cdot E{S}_2+\dots + dosag{e}_N\cdot E{S}_N}{\mathrm{mean}\left(E{S}_{1, \dots, N}\right)} $$

where ES is the effect size of the reported SNP–lipid association.

The PSs were scaled to have mean 0 and standard deviation 1. The association of the PS with lipid levels was confirmed using a meta-analysis of the outcomes of a model similar to the model described in Eq. 1 with PS as outcome instead of DNA methylation.

MR analysis was done using *AER* [61]. For each cohort a two-stage least-squares model (Eq. 4 and Eq. 5), a model suitable for epidemiological studies [12], was fitted per CpG followed by a meta-analysis of the results.

Stage 1:

4 $$ predlipi{d}_i={\gamma}_0+{\gamma}_1\cdot P{S}_i+{\gamma}_2\cdot gende{r}_i+{\gamma}_3\cdot ag{e}_i+{\gamma}_4\cdot \% mon{o}_i+{\gamma}_5\cdot \% lymp{h}_i+{\gamma}_6\cdot \%neu{t}_i+{\gamma}_7\cdot bisplat{e}_i+{\gamma}_8\cdot positio{n}_i + {\nu}_i\ \left(i=1,\dots, 3296\right) $$

Stage 2:

5 $$ DNA{m}_i={\beta}_0+{\beta}_1\cdot predlipi{d}_i+{\beta}_2\cdot gende{r}_i+{\beta}_3\cdot ag{e}_i+{\beta}_4\cdot \% mon{o}_i+{\beta}_5\cdot \% lymp{h}_i+{\beta}_6\cdot \%neu{t}_i+{\beta}_7\cdot bisplat{e}_i+{\beta}_8\cdot positio{n}_i+{\varepsilon}_i\left(i=1,\dots, 3296\right) $$

where predlipid is the predicted lipid level (TG, LDL-C, HDL-C) based on its polygenic score.

Power calculations for two-stage least-squares-based MR studies [15] were performed using the R implementation (https://github.com/kn3in/mRnd).

To address whether the PS SNPs directly affect DNA methylation as a *cis*-methylation QTL effect instead of through an effect on lipid levels, genotypes of SNPs (as dosage) within 1 Mb of CpGs resulting from the MR analysis were included as an additional covariate in the MR model (Eq. 4 and Eq. 5).

To test whether DNA methylation affected lipid levels, instead of lipid levels affecting DNA methylation (the direction of the effect evaluated in the PS-based MR analysis), SNPs were identified that were *cis*-methylation QTLs of CpGs associated with lipid levels found using the model defined by Eq. 6:

6 $$ DNA{m}_i={\beta}_0+{\beta}_1\cdot dosag{e}_i+{\beta}_2\cdot gende{r}_i+{\beta}_3\cdot ag{e}_i+{\beta}_4\cdot \% mon{o}_i+{\beta}_5\cdot \% lymp{h}_i+{\beta}_6\cdot \%neu{t}_i+{\beta}_7\cdot bisplat{e}_i+{\beta}_8\cdot positio{n}_i+{\varepsilon}_i\left(i=1,\dots, 3296\right) $$

Subsequently, the dosage of these meQTL SNPs was entered into the MR model as an instrument for DNA methylation (Eq. 7 and Eq. 8) and the results were meta-analyzed.

Stage 1:

7 $$ predDNA{m}_i={\gamma}_0+{\gamma}_1\cdot meqt{l}_i+{\gamma}_2\cdot gende{r}_i+{\gamma}_3\cdot ag{e}_i+{\gamma}_4\cdot \% mon{o}_i+{\gamma}_5\cdot \% lymp{h}_i+{\gamma}_6\cdot \%neu{t}_i+{\gamma}_7\cdot bisplat{e}_i+{\gamma}_8\cdot positio{n}_i + {\nu}_i\ \left(i=1,\dots, 3296\right) $$

Stage 2:

8 $$ lipi{d}_i={\beta}_0+{\beta}_1\cdot predDNA{m}_i+{\beta}_2\cdot gende{r}_i+{\beta}_3\cdot ag{e}_i+{\beta}_4\cdot \% mon{o}_i+{\beta}_5\cdot \% lymp{h}_i+{\beta}_6\cdot \%neu{t}_i+{\beta}_7\cdot bisplat{e}_i+{\beta}_8\cdot positio{n}_i+{\varepsilon}_i\left(i=1,\dots, 3296\right) $$

where predDNAm is the predicted DNA methylation level based on its methylation QTL and meqtl is the dosage of the methylation QTL.

To account for pleiotropic effects (as SNPs in the PSs are often associated with multiple lipids), multivariable instrumental variable analysis was done using the three PSs and three lipids in a two-stage least-squares model similar to the model defined by Eq. 4 and Eq. 5, followed by a meta-analysis of the results. This method performs well for lipid pleiotropy correction [27]. We compared the multivariable MR approach with an approach based on Egger regression [17]. For the association between lipids and PS SNPs required for Egger regression we used the estimates reported in a GWAS [11] and for the association between PS SNPs and DNA methylation we used Eq. 9. We combined the results in a meta-analysis.

9 $$ DNA{m}_i={\gamma}_0+{\gamma}_1\cdot dosag{e}_i+{\gamma}_2\cdot gende{r}_i+{\gamma}_3\cdot ag{e}_i+{\gamma}_4\cdot \% mon{o}_i+{\gamma}_5\cdot \% lymp{h}_i+{\gamma}_6\cdot \%neu{t}_i+{\gamma}_7\cdot bisplat{e}_i+{\gamma}_8\cdot positio{n}_i + {\nu}_i\ \left(i=1,\dots, 3296\right) $$

DNA methylation was associated with expression of genes within 100 kb in *limma* [57] using the linear model defined by Eq. 10 per cohort, after which the results were meta-analyzed.

10 $$ Expressio{n}_i={\beta}_0+{\beta}_1\cdot DNA{m}_i+{\beta}_2\cdot gende{r}_i+{\beta}_3\cdot ag{e}_i+{\beta}_4\cdot \% mon{o}_i+{\beta}_5\cdot \% lymp{h}_i+{\beta}_6\cdot \%neu{t}_i+{\beta}_7\cdot bisplat{e}_i+{\beta}_8\cdot positio{n}_i+ flowcel{l}_i+{\varepsilon}_i\left(i=1,\dots, 2044\right) $$

where flowcell is the Hiseq2000 flowcell.

## Additional files

Additional file 1: Full list of authors of the BIOS Consortium. (PDF 68 kb) Additional file 2: Quantile–quantile plots depicting the associations between TG, LDL-C, and HDL-C and genome-wide DNA methylation when compared with a uniform distribution expected by chance for a uncorrected meta-analysis z-values and b meta-analysis z-values corrected for genome-wide inflation using genomic control (λ = 1.0). (PDF 14800 kb) Additional file 3: Estimates and 95 % confidence intervals for the EWAS-identified CpGs for each cohort contributing to the meta-analysis for TG, LDL-C, and HDL-C. (PDF 297 kb) Additional file 4: The EWAS effect size estimates were not sensitive to the exclusion of non-fasted samples, the exclusion of samples with imputed cell counts, or the addition of current smoking behavior, lipid-lowering medication, or BMI as covariates. (PDF 273 kb) Additional file 5: The lipid-associated SNPs used to construct the polygenic scores. (CSV 1 kb) Additional file 6: Polygenic scores constructed from lipid GWAS-identified SNPs associate strongly with their respective lipid levels but explain only a small amount of their variance. (CSV 104 bytes) Additional file 7: The HDL-C PS is nominally associated with confounder neutrophil counts. (CSV 202 bytes) Additional file 8: Mendelian randomization reveals an effect of lipid levels on DNA methylation for some of the EWAS-identified CpGs. Estimate is the percentage change in DNA methylation per standard deviation change in lipid levels. (CSV 1 kb) Additional file 9: Comparison between EWAS and MR estimates (with 95 % confidence intervals). (PDF 109 kb) Additional file 10: Power calculations were performed for the situations where the EWAS effect size estimate is (1) the true causal effect size, (2) half the true causal effect size, and (3) double the true causal effect size. (CSV 1 kb) Additional file 11: Effect of lipid levels on DNA methylation corrected for nearest PS SNP within 1 Mb. Estimate is the percentage change in DNA methylation per standard deviation change in lipid levels. (CSV 289 bytes) Additional file 12: Association between DNA methylation and genetic variation for the CpGs and their strongest associating SNP within 100 kb. (CSV 1 kb) Additional file 13: No effect of DNA methylation on lipid levels was found using the strongest associating SNP within 100 kb for each CpG as an instrument. Estimate is the standard deviation change in lipid levels per percentage change in DNA methylation. (CSV 1 kb) Additional file 14: DNA methylation was associated with gene expression. (PDF 21974 kb)
