# Supplementary material for: Blood lipids influence DNA methylation in circulating cells
Source: Genome Biol. 2016 Jun 27;17:138. doi: 10.1186/s13059-016-1000-6 (PMC4922056; doi:10.1186/s13059-016-1000-6)
Supplement: Additional file 2: — Quantile–quantile plots depicting the associations between TG, LDL-C, and HDL-C and genome-wide DNA methylation when compared with a uniform distribution expected by chance for a uncorrected meta-analysis z-values and b meta-analysis z-values corrected for genome-wide inflation using genomic control (λ = 1.0). (PDF 14800 kb) [file 13059_2016_1000_MOESM2_ESM.pdf]

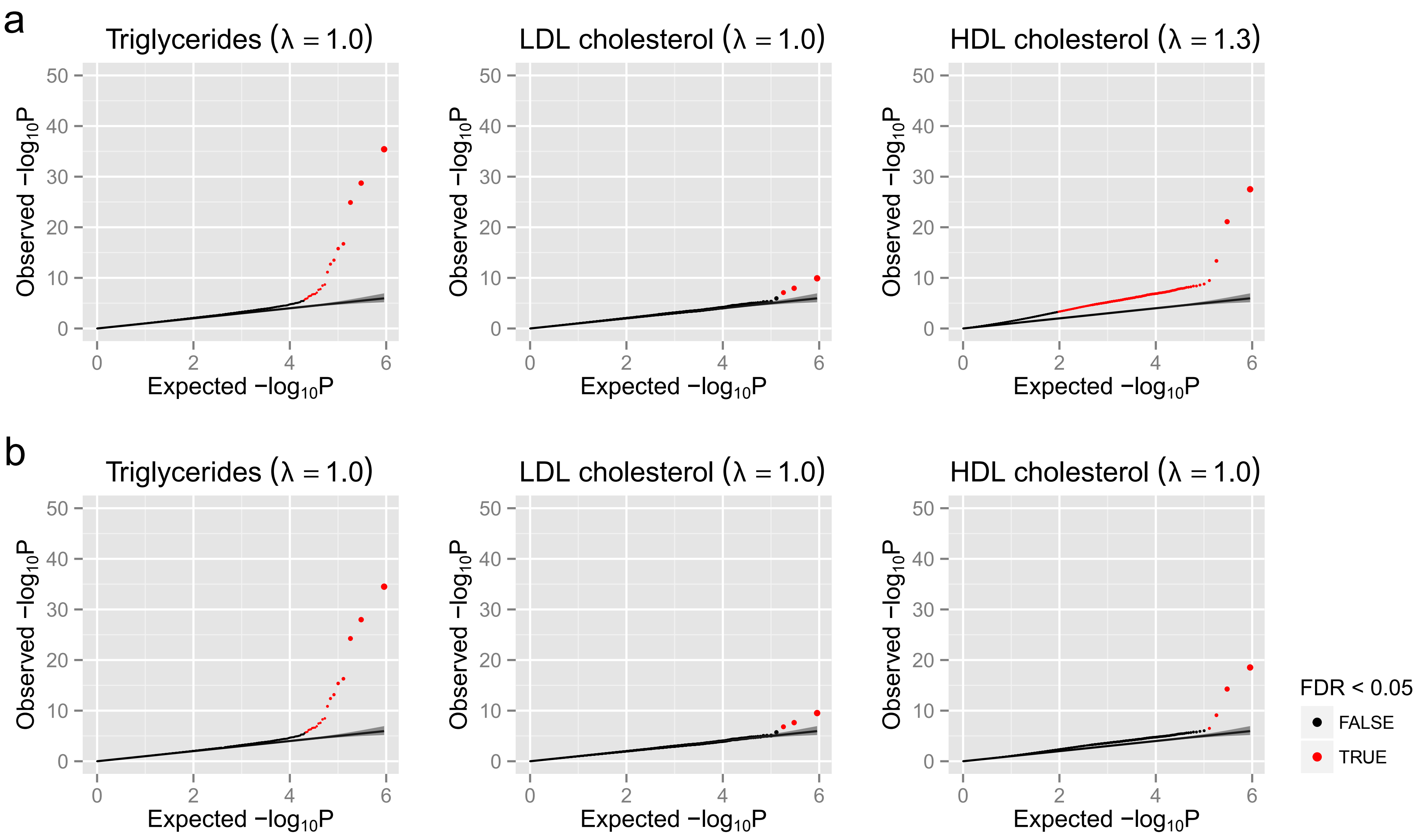

a
Triglycerides (λ = 1.0)
LDL cholesterol (λ = 1.0)
HDL cholesterol (λ = 1.3)
b
Triglycerides (λ = 1.0)
LDL cholesterol (λ = 1.0)
HDL cholesterol (λ = 1.0)
Observed −log10P
Expected −log10P
0
10
20
30
40
50
0
2
4
6
FDR < 0.05
FALSE
TRUE
