# Supplementary figures and images for: Blood lipids influence DNA methylation in circulating cells
Source: Genome Biol. 2016 Jun 27;17:138. doi: 10.1186/s13059-016-1000-6 (PMC4922056; doi:10.1186/s13059-016-1000-6)

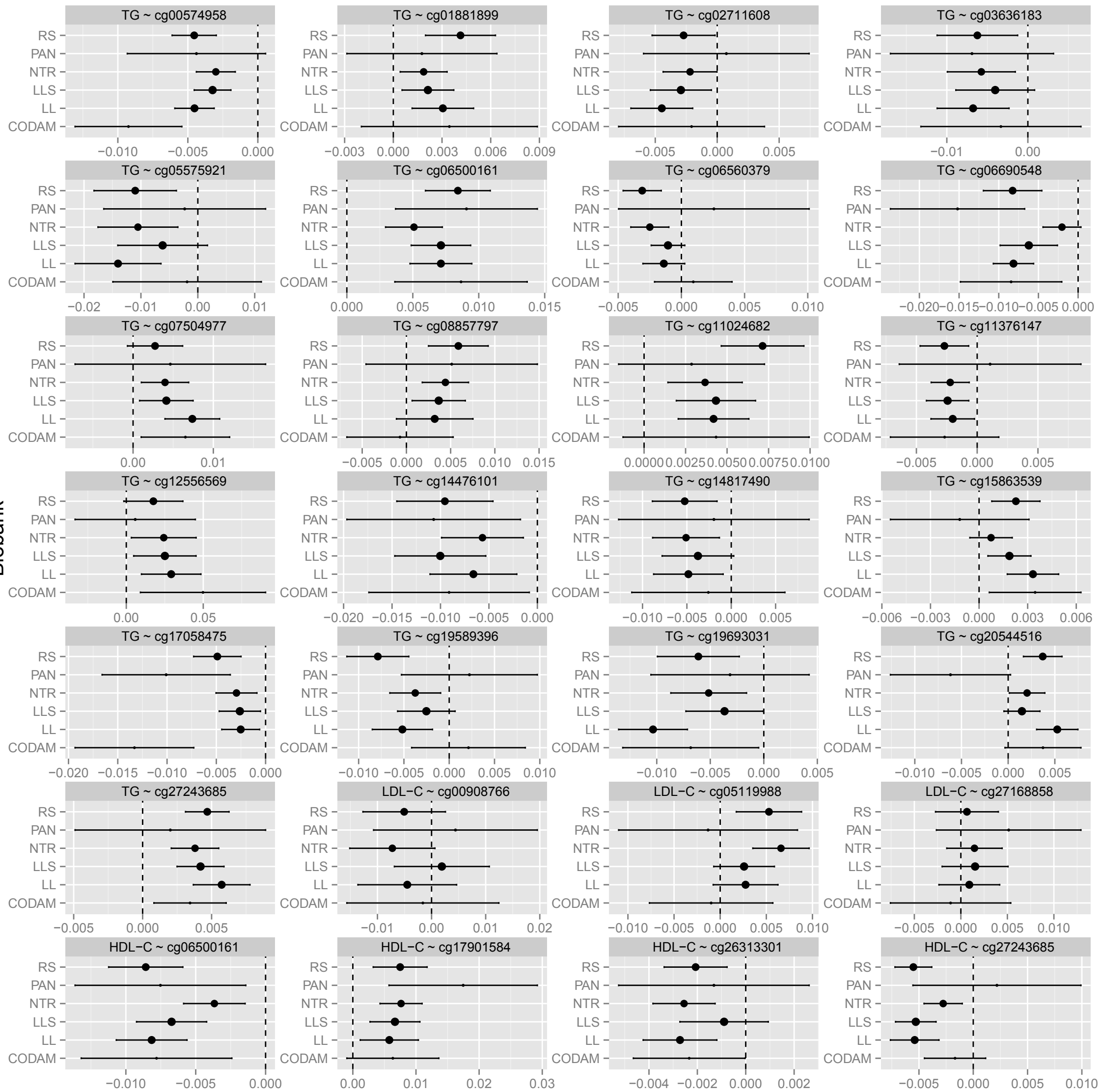

Supplement: Additional file 3: — Estimates and 95 % confidence intervals for the EWAS-identified CpGs for each cohort contributing to the meta-analysis for TG, LDL-C, and HDL-C. (PDF 297 kb) [file 13059_2016_1000_MOESM3_ESM.pdf]

## TG

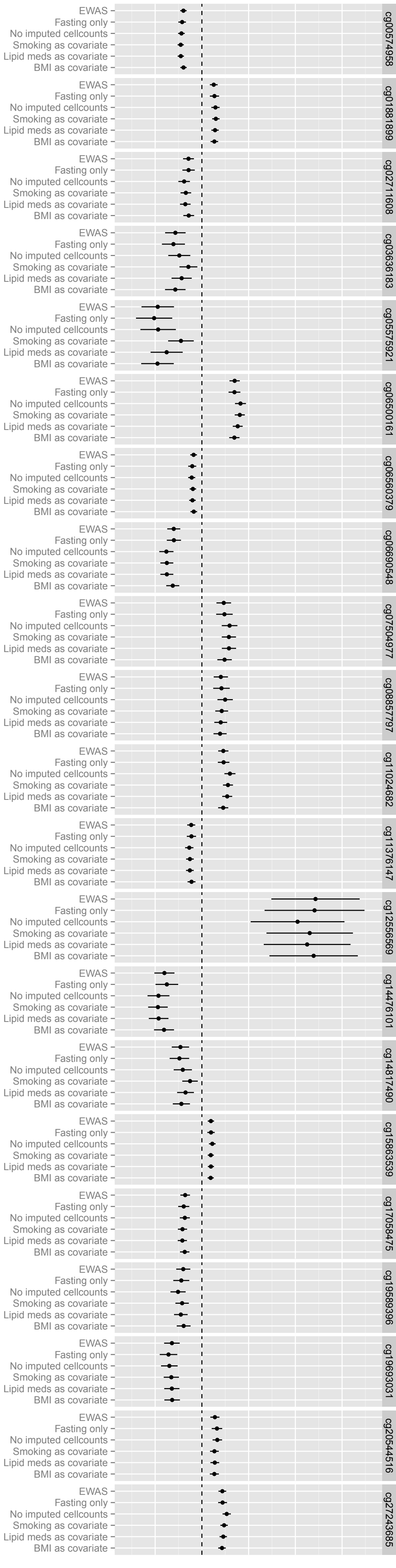

## LDL-C

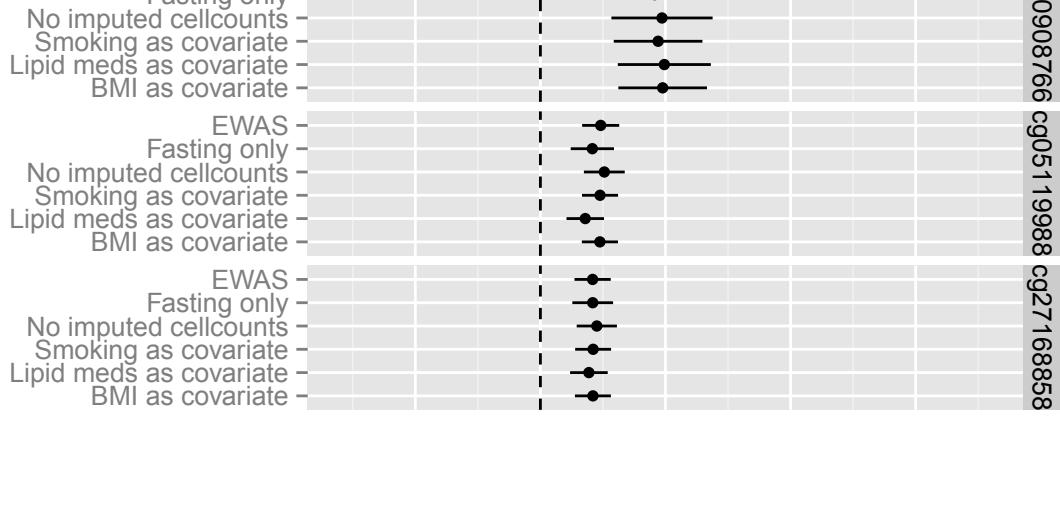

## HDL-C

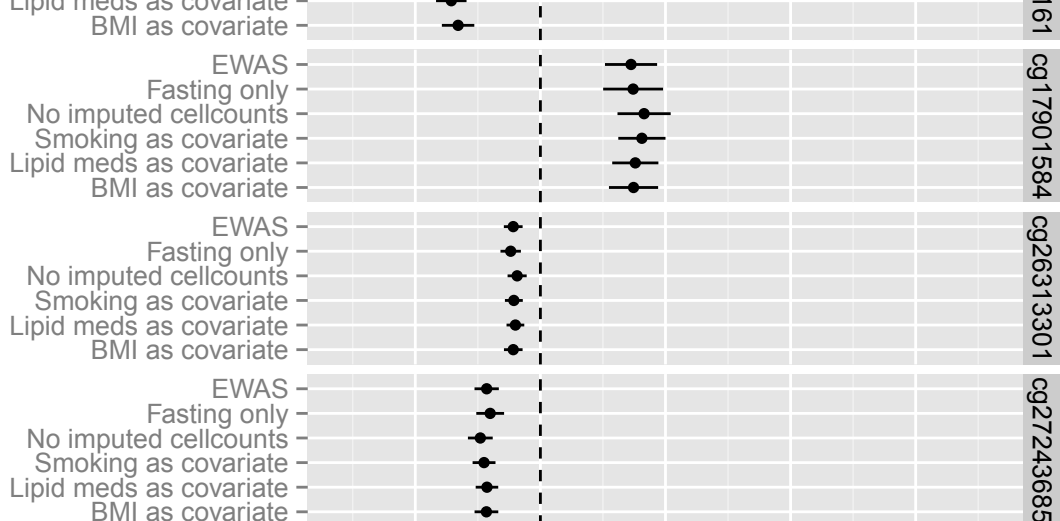

-0.01

0.00

0.01

0.02

0.03

Estimate

Supplement: Additional file 4: — The EWAS effect size estimates were not sensitive to the exclusion of non-fasted samples, the exclusion of samples with imputed cell counts, or the addition of current smoking behavior, lipid-lowering medication, or BMI as covariates. (PDF 273 kb) [file 13059_2016_1000_MOESM4_ESM.pdf]

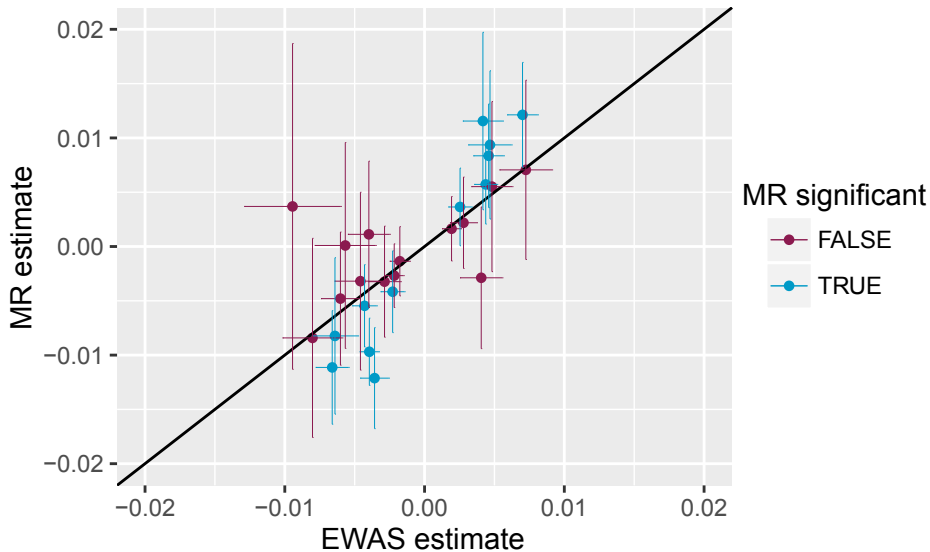

Supplement: Additional file 9: — Comparison between EWAS and MR estimates (with 95 % confidence intervals). (PDF 109 kb) [file 13059_2016_1000_MOESM9_ESM.pdf]

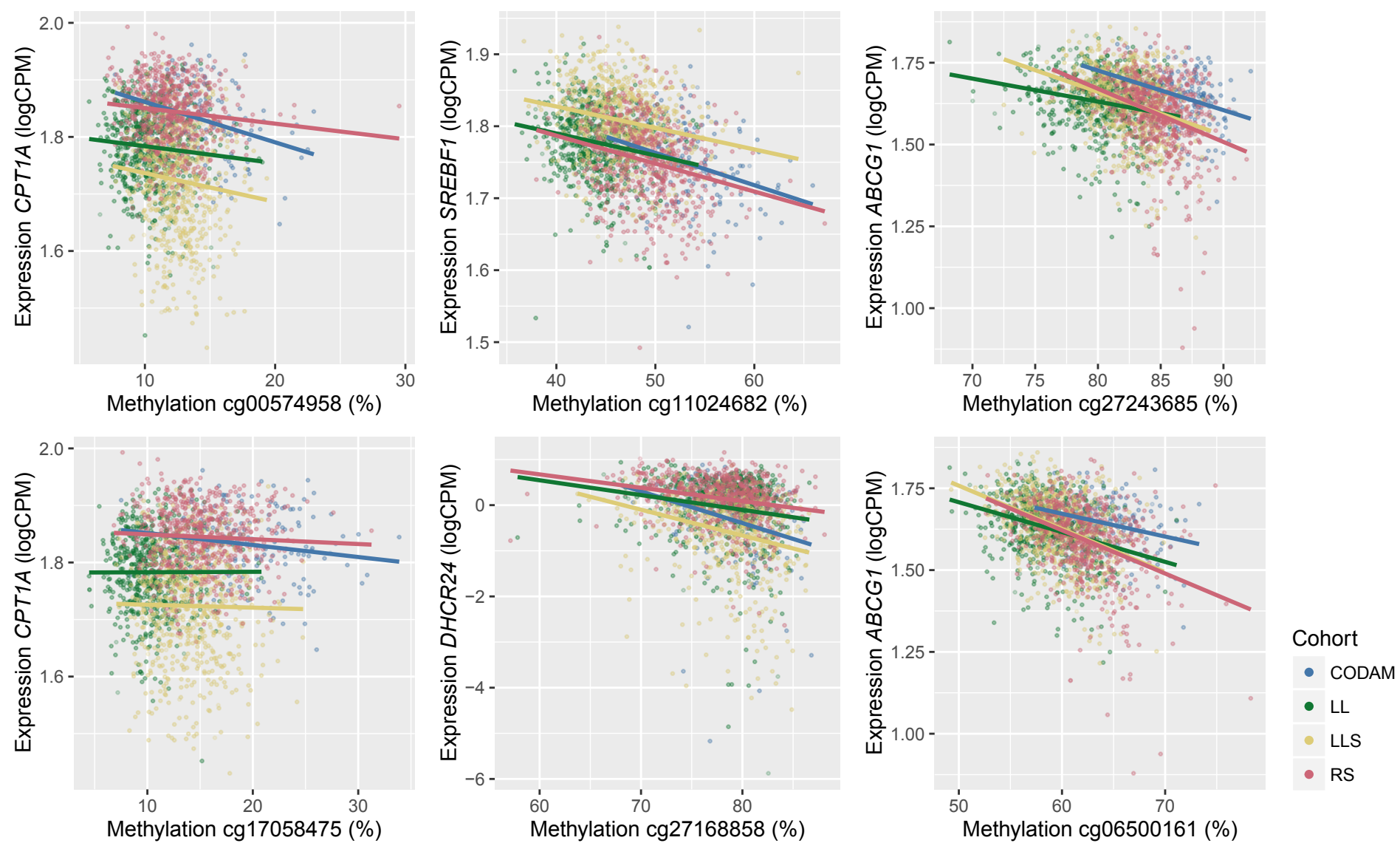

Supplement: Additional file 14: — DNA methylation was associated with gene expression. (PDF 21974 kb) [file 13059_2016_1000_MOESM14_ESM.pdf]
